# Factors associated with the delay in informed consent procedures of patients with ST-segment elevation myocardial infarction and its influence on door-to-balloon time: a nationwide retrospective cohort study

**DOI:** 10.2478/jtim-2023-0127

**Published:** 2024-03-21

**Authors:** Mailikezhati Maimaitiming, Junxiong Ma, Xuejie Dong, Shuduo Zhou, Na Li, Zheng Zhang, Shijuan Lu, Lianglong Chen, Likun Ma, Bo Yu, Yitong Ma, Xingsheng Zhao, Zhaofen Zheng, Hong Shi, Zhijie Zheng, Yinzi Jin, Yong Huo

**Affiliations:** Department of Global Health, School of Public Health, Peking University, Beijing, China; Institute for Global Health and Development, Peking University, Beijing, China; First People’s Hospital of Lanzhou University, Lanzhou, Gansu Province, China; Haikou People’s Hospital, Haikou, Hainan Province, China; Fujian Medical University Union Hospital, Fuzhou, Fujian Province, China; Anhui Provincial Hospital, Hefei, Anhui Province, China; Second Affiliated Hospital of Harbin Medical University, Harbin, Heilongjiang Province, China; First Affiliated Hospital of Xinjiang Medical University, Urumqi, Xinjiang Uygur Autonomous Region, China; Inner Mongolia People’s Hospital, Huhhot, Inner Mongolia Autonomous Region, China; Hunan Provincial People’s Hospital, Changsha, Hunan Province, China; Chinese Medical Association, Beijing, China; Department of Cardiology, Peking University First Hospital, Beijing, China

**Keywords:** informed consent delay, ST-segment elevation myocardial infarction, percutaneous coronary intervention, door-to-balloon time

## Abstract

**Background and Objectives:**

ST-segment elevation myocardial infarction (STEMI) is the deadliest and most time-sensitive acute cardiac event. However, failure to achieve timely informed consent is an important contributor to in-hospital delay in STEMI care in China. We investigated the factors associated with informed consent delay in patients with STEMI undergoing percutaneous coronary intervention (PCI) and the association between the delay and door-to-balloon time.

**Methods:**

We conducted a nationally representative retrospective cohort study using patient data reported by hospital-based chest pain centers from 1 January 2016 to 31 December 2020. We applied generalized linear mixed models and negative binomial regression to estimate factors independently predicting informed consent delay time. Logistic regressions were fitted to investigate the association of the informed consent delay time and door-to-balloon time, adjusting for patient characteristics.

**Results:**

In total, 257, 510 patients were enrolled in the analysis. Mean informed consent delay time was 22.4 min (SD = 24.0), accounting for 39.3% in door-to-balloon time. Older age (≥65 years) was significantly correlated with informed consent delay time (RR: 1.034, *P* = 0.001). Compared with ethnic Han patients, the minority (RR: 1.146, *P* < 0.001) had more likelihood to extend consent giving; compared with patients who were single, longer informed consent time was found in married patients (RR: 1.054, *P* = 0.006). Patients with intermittent chest pain (RR: 1.034, *P* = 0.011), and chest pain relief (RR: 1.085, *P* = 0.005) were more likely to delay informed consent. As for transfer modes, EMS (RR: 1.063, *P* < 0.001), transfer-in (RR: 1.820, *P* < 0.001), and in-hospital onset (RR: 1.099, *P* = 0.002) all had positive correlations with informed consent delay time compared to walk-in. Informed consent delay was significantly associated with prolonged door-to-balloon time (OR: 1.002, *P* < 0.001).

**Conclusion:**

Informed consent delay is significantly associated with the door-to-balloon time which plays a crucial role in achieving better outcomes for patients with STEMI. It is essential to shorten the delay time by identifying and intervening modifiable factors that are associated with shortening the informed consent procedure in China and other countries.

## Introduction

ST-segment elevation myocardial infarction (STEMI) is the deadliest cardiovascular event and accounts for an estimated 50% of ischemic heart disease, and it is also the second leading cause of death in China.^[1]^ The latest estimation revealed that the national rates of hospital admission for STEMI per 100, 000 population increased from 3.7 in 2001 to 15.8 in 2011. STEMI-associated mortality more than doubled during the past three decades, and this trend is predicted to accelerate, imposing a surging burden on individuals, communities, and health systems.^[2]^ According to clinical guidelines,^[3]^ percutaneous coronary intervention (PCI) is the optimal treatment for STEMI, and its effect mostly correlates with the door-to-balloon time. It is recommended to control the door-to-balloon time to within 90 min as an important index of in-hospital delay.^[4,5,6]^ The door-to-balloon time is estimated to account for most of the treatment delay in China,^[7,8]^ and only about 10% of patients receive timely PCI therapy.^[9]^ Quite commonly, informed consent delay is the most important contributor to prolonged in-hospital delay in China. The conversation regarding informed consent is usually not completed until after the catheterization laboratory is ready for PCI, which results in delayed treatment and poor clinical outcomes.^[8,10,11]^

Undoubtedly, informed consent is indispensable for well- organized medical practice. Generally, before performing any surgery, healthcare professionals are required to obtain consent from capable patients or their legal surrogate decision-makers.^[11,12]^ The purpose of informed consent is to create an open and honest working relationship between the patients and physicians in which the benefits and risks of proposed treatment are discussed. This empowers patients with autonomy and liberty to make clinical decisions. Although reducing in-hospital delay by improving the informed consent process is of the essence, it is also appropriate to spend adequate time to obtain consent from patients in consideration of their disease severity and education level as well as the need to have a discussion with their family.^[13,14]^ Nevertheless, in contrast to usual medical practice, decisions must be made urgently in emergency care to improve the effect of medical treatment for patients in critical situations. During the provision of emergency care, several challenges can affect the process of informed consent, such as the importance of very narrow therapeutic windows, the inability of patients to provide informed consent, or the absence of substitute decision-makers.^[15]^

In China, healthcare practitioners are required to obtain informed consent from patients or relatives prior to emergency surgery, although Chinese Civil Code indicates that it is legal to give treatment to a person without informed consent on the premise that the person is in life-threatening condition and it is impossible to obtain the person’s or a relative’s consent.^[12]^ Notably, in contrast to Western law, patients’ families are granted the same right to decide as that granted to the patients themselves and sometimes even play a dominant role in China.^[16]^ Regardless of the severity and urgency of the disease, physicians commonly do not start the surgical procedure unless the patient and his or her relatives reach an agreement.

In many situations, the informed consent procedure in China is rather long in duration, leading to longer in-hospital delays. The patient’s condition may deteriorate with this prolongation, posing challenges to emergency surgery and negatively impacting the patient’s clinical outcome. This phenomenon is critically serious among patients with STEMI, which is the most time-sensitive acute cardiac event. According to the standardized diagnosis and treatment process, patients with STEMI who are transported by emergency medical services (EMS) should undergo an electrocardiographic examination in the ambulance. The results can be transferred to the emergency department, and the coronary care unit should then rapidly make a preliminary diagnosis based on these results and maintain real-time communication with the ambulance. Thus, the informed consent conversation usually begins on the ambulance before the patient arrives at the hospital. In China, however, the use of EMS among patients with STEMI is rather low.^[9]^ Additionally, regardless of whether they are transported by EMS, patients and their families always spend a long time becoming familiar with the severity of the patient’s disease condition and understanding the therapy, and they are often unable to establish trust with healthcare professionals in a short time,^[17]^ resulting in long delays in obtaining written informed consent. Thus, the informed consent procedure is often still incomplete when the catheterization laboratory is ready for activation, resulting in prolonged in-hospital delay. Nevertheless, the delay in the informed consent procedure is modifiable, and reducing the time taken to obtain informed consent requires an understanding of the modifiable factors that are involved.

Prior studies have mainly focused on the whole timeline of treatment delay in STEMI care and factors related to the door-to-balloon time.^[5,18,19]^ Although some studies have investigated the process of informed consent, most of them qualitatively explored the approaches to improving this process,^[17,20,21,22]^ and qualitative studies examined the obtainment of informed consent during a clinical trial instead of surgery.^[15,23]^ To the best of our knowledge, no studies have been conducted to identify the causes of delay in obtaining informed consent or to examine whether the delay is associated with the door-to-balloon time in China and other countries with similar sociocultural backgrounds where it is required to obtain informed consent prior to emergency surgery.

To fill these research gaps, the present study was performed to investigate the factors influencing the delay in the informed consent procedure for patients with STEMI undergoing PCI and the association of this delay with the door-to-balloon time. This nationally representative retrospective cohort study was performed using patient data obtained from all of the chest pain centers in China. We anticipated identifying factors that have not been reported to be associated with shortening the interval for informed consent, thus contributing to reducing emergency treatment delay in China and other countries with similar sociocultural contexts.

## Methods

### Data collection and participants

Data were extracted from the China Chest Pain Center Database of the Chinese Cardiovascular Association (http://data.chinacpc.org/), which is a nationwide web-based unified database that collects data of patients discharged from hospital-based chest pain centers.^[24]^ Cases should be reported to the Database by each chest pain center according to the data elements abstracted from medical charts, including patient demographics, pre-hospital symptoms and transport mode, vital signs at presentation, in-hospital therapy, clinical outcomes, and diagnosis at discharge.

In this study, we included data reported from 1 January 2016 to 31 December 2021. Participants eligible for the study (1) were confirmed to have STEMI, (2) received reperfusion therapy by PCI, and (3) were aged 18 years or older. We excluded participants whose medical records had failed to report the time points, discharge diagnosis, and selected factors that influenced the informed consent delay. In total, 257, 510 participants were enrolled for the final analysis.

### Measurement

#### Informed consent delay time

The informed consent delay time was defined as the interval between the decision to perform PCI and completion of the informed consent signature. Once PCI therapy has been recommended, the conversation between physicians and patients begins, indicating that the informed consent procedure has also begun. Completion of the signature is a signal to prepare for activation of the catheterization laboratory. We also calculated the proportion of the informed consent delay time within the door-to-balloon time, which was the sum of the time intervals from arrival at the hospital to the PCI decision, from the PCI decision to the informed consent signature, and from the informed consent signature to intracoronary balloon inflation.

#### Factors associated with informed consent delay time

The variables that were assumed to have an association with the informed consent delay time were (1) patient demographics, including sex, age, ethnicity, employment, education level, marriage status, insurance, and comorbidities;(2) pre-hospital symptoms, including sustainable chest pain, intermittent chest pain, and chest pain relief;(3) vital signs at presentation, including the respiratory rate, heart rate, blood pressure, and Killip class (class I, II, III, and IV corresponding to no, mild, moderate and severe symptoms, respectively); and (4) transport modes (classified as EMS transport, transfer-in, walk-in, and in-hospital onset). These factors were also controlled as covariates to predict the association between the informed consent delay time and D2B time.

### Statistical analysis

We examined the informed consent delay time and its proportion of the door-to-balloon time of the patients. Continuous variables are expressed as mean and standard deviation (SD), and categorical variables are presented as frequency and percentage. To account for clustering of patients within hospitals, we employed generalized linear mixed models with a random-effect term for hospitals to examine patient-related factors independently contributing to the informed consent delay time. A negative binomial regression analysis was performed for the informed consent delay time, and the effect estimates are reported as the relative risk (RR) and 95% confidence interval (CI). Logistic regression was fitted to investigate the association between the informed consent delay time and the door- to-balloon time, with the effect estimates reported as the odds ratio (OR) and 95% CI. All analyses were performed by R software Version 3.6.3 (R Foundation for Statistical Computing, Vienna, Austria). A *P* value of < 0.05 was considered statistically significant.

## Results

### Patient characteristics

**Figure 1:**
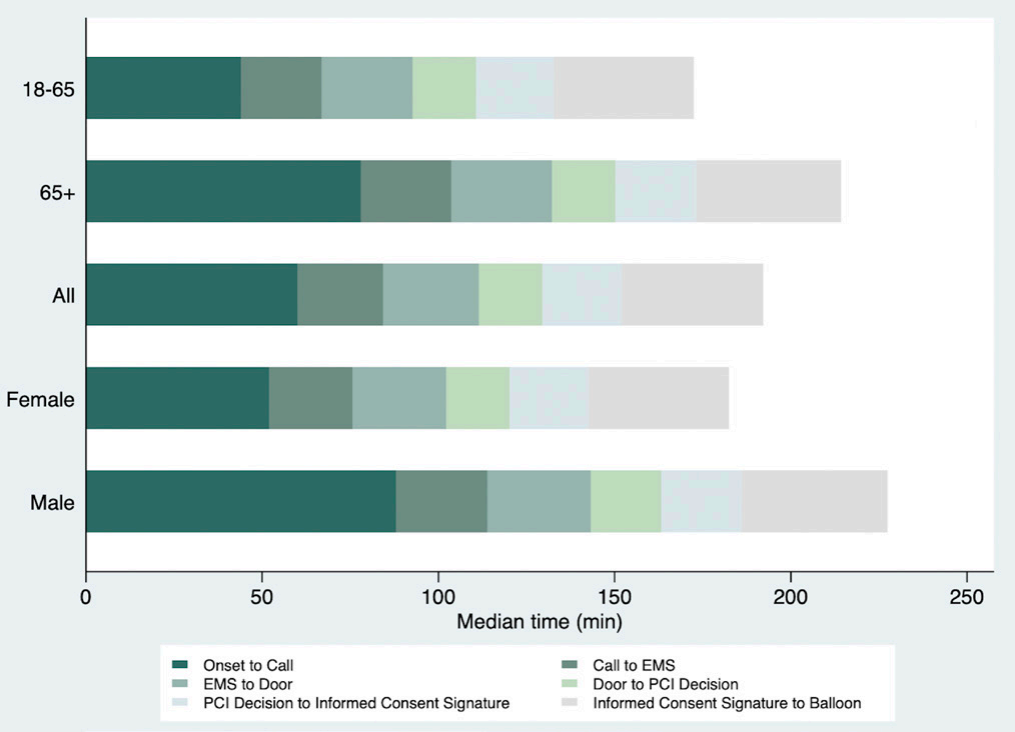
Distribution of treatment time among patients with STEMI. The figure shows the key time intervals among patients in the total cohort, male and female patients, and patients aged 18–65 and >65 years. The key time intervals were calculated according to specific time points and included the onset-to-call time (only for patients who called EMS), call-to-EMS time (only for patients who called EMS), EMS-to-door time (only for patients who called EMS), door-to-PCI decision time, PCI decision-to-informed consent signature time, and informed consent signature-to-balloon time. Notably, the door-to-balloon time was the sum of the door-to-PCI decision time, PCI decision-to-informed consent signature time, and informed consent signature- to-balloon time.

In total, 257, 510 participants were finally enrolled in the study, of whom 75.3% (193, 825) were male and 52.5% were from 18 to 65 years of age. The patient characteristics are listed in Table 1. Among all participants, the mean informed consent delay time was 22.4 min (SD = 24.0), accounting for 39.3% of the door-to-balloon time. The longest informed consent delay time (32.1 min, SD = 32.1) was found in patients who were transferred from other hospitals, whereas patients who came to hospitals by themselves had the shortest delay time (18.0 min, SD = 18.0). The distributions of the treatment time from onset to intracoronary balloon inflation by sex and age are shown in Figure 1.

**Table 1:** Patient characteristics

Characteristics	*n* (%)	Informed consent delayed time, minutes mean (SD)	*P*	Percentage in D2B, % mean (SD)	*P*
Admissions for STEMI	257510	22.4 (24.0)	-	39.3 (64.3)	-
Sex					
Male	193825 (75.3)	22.3 (24.0)	0.006	39.5 (64.7)	0.152
Female	63684 (24.7)	22.8 (24.2)		38.8 (62.7)	
Age (years)					
18-65	135271 (52.5)	22.0 (23.6)	<0.001	39.0 (63.7)	0.037
65+	122239 (47.5)	23.0 (24.6)		39.8 (65.1)	
Ethnicity					
Ethnic Han	12464 (7.0)	27.8 (31.3)	<0.001	53.4 (90.9)	<0.001
Minority	165236 (93.0)	23.0 (24.2)		40.6 (65.2)	
Employment status					
Unemployed	30081 (24.6)	22.5 (23.5)	<0.001	37.8 (61.3)	<0.001
Employed	92059 (75.4)	23.6 (25.4)		41.8 (67.6)	
Marriage status					
Single	139629 (92.9)	23.6 (25.1)	0.176	42.4 (68.6)	0.221
Married	10673 (7.1)	24.1 (24.9)		41.0 (64.3)	
Health Insurance					
UEBMI	31285 (24.0)	21.7 (23.1)	<0.001	35.4 (57.3)	<0.001
NRCMS	50931 (39.1)	23.2 (24.8)		42.1 (67.1)	
URBMI	38698 (29.7)	24.2 (25.8)		43.5 (69.9)	
None	9272 (7.1)	23.6 (24.3)		40.6 (61.6)	
Symptoms at presentation					
Sustainable chest pain	181880 (74.6)	22.4 (24.0)	<0.001	40.1 (65.4)	<0.001
Intermittent chest pain	50008 (20.5)	22.2 (23.9)		34.8 (57.7)	
Chest pain relief	11982 (4.9)	26.4 (28.3)		47.2 (76.5)	
Comorbidity					
No	29484 (12.0)	21.7 (23.3)	<0.001	39.2 (64.6)	0.970
Yes	217072 (88.0)	22.5 (24.1)		39.2 (64.1)	
Heart rate (beats/min)					
60-100	188520 (73.4)	22.6 (24.4)	<0.001	40.3 (66.2)	<0.001
<60 or > 100	68145 (26.6)	21.9 (23.0)		36.3 (58.0)	
High blood pressure					
No	189328 (73.5)	22.8 (24.5)	<0.001	41.5 (68.0)	<0.001
Yes	68182 (26.5)	21.3 (22.6)		33.4 (52.6)	
Killip class					
I	183094 (75.1)	22.0 (23.5)	<0.001	38.6 (62.6)	<0.001
II	35347 (14.5)	24.9 (27.0)		45.0 (76.9)	
III	10611 (4.4)	23.1 (24.6)		35.0 (52.6)	
IV	14754 (6.1)	24.0 (25.2)		40.7 (63.9)	
Transport modes					
EMS	28590 (11.1)	19.1 (18.0)	<0.001	33.0 (36.7)	<0.001
Transfer-in	67433 (26.2)	32.1 (32.1)		77.7 (92.8)	
Walk-in	155105 (60.2)	18.0 (18.0)		20.6 (15.7)	
In-hospital onset	6358 (2.5)	20.7 (20.9)		-	

SD: standard deviation; D2B: door-to-balloon time; STEMI: ST-segment elevation myocardial infarction; UEBMI: Urban Employee Basic Medical Insurance; NRCMS: New Rural Cooperative Medical Scheme; URBMI: Urban Resident Basic Medical Insurance; EMS: emergency medical services.

### Predictors of informed consent delay time

Table 2 shows the factors that independently contributed to the informed consent delay time in patients with STEMI. Elderly patients (age of ≥65 years) (RR: 1.034, 95% CI: 1.014–1.055) were more likely to have prolonged informed consent. Compared with patients of Han ethnicity, patients of ethnic minorities (RR: 1.146, 95% CI: 1.104–1.190) were also more likely to have prolonged informed consent. An employed status (RR: 0.975, 95% CI: 0.953–0.997) had a negative association with the informed consent delay time. A longer informed consent time was found in married patients than in single patients (RR: 1.054, 95% CI: 1.016–1.094). In terms of medical insurance, Urban Resident Basic Medical Insurance (URBMI) (RR: 1.05, 95% CI: 1.027–1.074) was significantly more strongly associated with the informed consent delay time than New Rural Cooperative Medical Insurance Scheme (NRCMS)

**Table 2:** Negative binomial regression analysis predicting informed consent delay time

	RR	95% CI	*P*
Sex (reference: Male)	0.998	[0.975, 1.020]	0.832
Age (reference: 18-65)	1.034	[1.014, 1.055]	0.001
Ethnicity (reference: Ethnic Han)	1.146	[1.104, 1.190]	<0.001
Employment (reference: Unemployed)	0.975	[0.953, 0.997]	0.023
Marriage (reference: Single)	1.054	[1.016, 1.094]	0.006
Insurance (reference: NRCMS)			
UEBMI	1.017	[0.993, 1.042]	0.159
URBMI	1.050	[1.027, 1.074]	<0.001
None	1.036	[0.996, 1.077]	0.078
Symptoms at presentation (reference: Sustainable chest pain)			
Intermittent chest pain	1.034	[1.008, 1.060]	0.011
Chest pain relief	1.085	[1.025, 1.150]	0.005
Comorbidity (reference: None)	1.059	[1.026, 1.094]	<0.001
Heart rate (reference: 60-100)	0.989	[0.969, 1.011]	0.320
High blood pressure (reference: No)	0.993	[0.972, 1.013]	0.474
Killip class (reference: I )			
II	1.097	[1.068, 1.128]	<0.001
III	1.041	[0.984, 1.102]	0.166
IV	1.082	[1.037, 1.130]	<0.001
Transport modes (reference: Walk-in)			
EMS	1.063	[1.033, 1.094]	<0.001
Transfer-in	1.820	[1.783, 1.857]	<0.001
In-hospital onset	1.099	[1.036, 1.168]	0.002

RR: relative risk; 95% CI: 95% confidence interval; NRCMS: New Rural Cooperative Medical Scheme; UEBMI: Urban Employment Basic Medical Insurance; URBMI: Urban Resident Basic Medical Insurance; EMS: emergency medical services.

Compared with sustainable chest pain, intermittent chest pain (RR: 1.034, 95% CI: 1.008–1.060) and chest pain relief (RR: 1.085, 95% CI: 1.025–1.150) were positively associated with the informed consent delay time. Patients who had comorbidities (RR: 1.059, 95% CI: 1.026–1.094), Killip class II (RR: 1.097, 95% CI: 1.068–1.128), and Killip class IV (RR: 1.082, 95% CI: 1.037–1.130) tended to have a longer informed consent time. As for transport modes, EMS transport (RR: 1.063, 95% CI: 1.033–1.094), transferin (RR: 1.820, 95% CI: 1.783–1.857), and in-hospital onset (RR: 1.099, 95% CI: 1.036–1.168) had positive correlations with the informed consent delay time compared with walk-in.

### Influence of informed consent delay on door-to- balloon time

**Figure 2:**
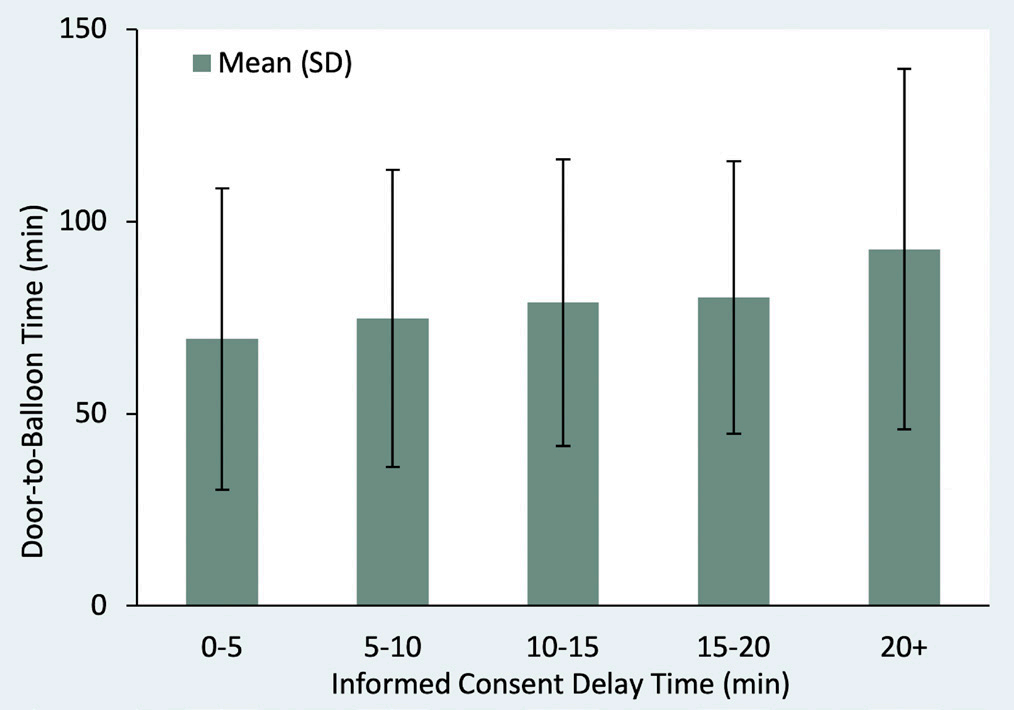
Distribution of door-to-balloon time with informed consent delay time among patients with STEMI. The figure shows the mean (SD) door-to-balloon time in participants with informed consent delay of 0–5 min, 5–10 min, 10–15 min, 15–20 min, and >20 min.

The association between informed consent delay and the door-to-balloon time is shown in Table 3. After controlling for covariates, an additional minute of the informed consent delay time was associated with a 0.2% longer door-to-balloon time (OR: 1.002, 95% CI: 1.002–1.003). Figure 2 shows the distribution of the door-to-balloon time with the informed consent delay time. Patients with an informed consent delay time of 0–5 min, 5–10 min, 10–15 min, 15–20 min, and > 20 min had a median door-to-balloon time of 69.4 (SD = 37.2) minutes, 70.8 (SD = 38.6) minutes, 74.9 (SD = 39.2) minutes, 80.2 (SD = 35.4) minutes, and 92.8 (SD = 46.9) minutes, respectively.

**Table 3:** Associations between informed consent delay and door-to-balloon time

	OR	95% CI	*P*
Informed consent delay time	1.002	[1.002, 1.003]	<0.001
Sex (reference: Male)	1.028	[1.014, 1.042]	<0.001
Age (reference: 18-65)	1.007	[0.995, 1.019]	0.257
Ethnicity (reference: Ethnic Han)	1.037	[1.013, 1.061]	0.002
Employment (reference: Employed)	1.013	[0.999, 1.026]	0.066
Marriage (reference: Single)	1.012	[0.990, 1.036]	0.291
Insurance (reference: NRCMS)			
UEBMI	1.017	[1.003, 1.032]	0.020
URBMI	1.005	[0.991, 1.019]	0.492
None	1.059	[1.035, 1.084]	<0.001
Symptoms at presentation (reference: Sustainable chest pain)			
Intermittent chest pain	1.101	[1.084, 1.118]	<0.001
Chest pain relief	1.127	[1.086, 1.169]	<0.001
Comorbidity (reference: None)	1.005	[0.986, 1.025]	0.613
Heart rate (reference: 60-100)	0.998	[0.985, 1.011]	0.741
High blood pressure (reference: No)	1.032	[1.019, 1.045]	<0.001
Killip class (reference: I )			
II	1.075	[1.057, 1.093]	<0.001
III	1.133	[1.093, 1.175]	<0.001
IV	1.061	[1.034, 1.090]	<0.001
Transport modes (reference: Walk-in)			
EMS	0.791	[0.778, 0.805]	<0.001
Transfer-in	0.679	[0.670, 0.687]	<0.001
In-hospital onset	-		-

OR: odds ratio; 95% CI: 95% confidence interval; NRCMS: New Rural Cooperative Medical Scheme; UEBMI: Urban Employment Basic Medical Insurance; URBMI: Urban Resident Basic Medical Insurance; EMS: emergency medical services.

## Discussion

In the context of China and other Asian countries, *e.g*., India, performing a complete informed consent procedure is necessary for any emergency.^[11,12]^ Although a prior study revealed the common occurrence of failure to provide timely informed consent in these countries,^[11]^ there is less evidence regarding the factors associated with informed consent delay, which restricts the potential to reduce delays in emergency treatment. Taking advantage of a nationally representative population-based retrospective cohort, we were able to identify significant factors associated with the informed consent delay time in patients with STEMI, including older age, minority ethnicities, unemployment, a married status, URBMI, atypical symptoms, comorbidities, and transfer modes of EMS, transfer-in, and in-hospital onset. Despite adjustment for covariates, we observed that prolongation of the informed consent procedure was positively associated with the door-to-balloon time.

We explored the potential mechanisms underlying the association between the significant factors and informed consent delay. We found that informed consent delay was more likely to occur in older patients, which is consistent with a previous study.^[19]^ Because of the special social culture in China, the major consent signers are not patients but their relatives.^[11]^ Even relevant laws and regulations do not stipulate that medical personnel should obtain consent from the patients themselves first.^[16]^ Instead, family members are commonly given equal or greater right to providing informed consent as that given the patients.^[16]^ As a deep-rooted system of philosophy, Confucianism subtly steers Chinese people to more highly value the opinions of the whole family than of the individual.^[12]^ Older patients usually come to the hospital with their relatives, and the relatives make decisions on behalf of the patients. However, it is generally difficult for family members to achieve consensus within a limited time,^[13,20]^ which influences the informed consent delay. This difficulty can be attributed to deteriorating doctor-patient relationships and other patient-related factors,^[17,25]^ such as a low socioeconomic level (*e.g*., low education attainment and unemployment). These are also the reason that married patients had a higher likelihood of deferring consent compared with single patients with STEMI. Moreover, older patients might have other comorbidities, causing their relatives to require a long time to assess the risk of treatment before making a decision. This is supported by our study in that patients with STEMI who had comorbidities were more likely to have informed consent delay.

Our results showed that patients of ethnic minorities were more likely to have delayed informed consent compared with ethnic Han patients. This is in line with a report from the American National Cardiovascular Data Registry, which found that treatment delays among non-English-speakers were caused by not providing consent in a timely manner because of culture bias and language barriers.^[19]^ Because most patients of ethnic minorities in China speak their own language rather than Mandarin, they might have difficulties in communicating with healthcare workers. Minority culture is also an indispensable factor that contributes to patients’ preferences and influences their decision-making.^[19]^ It is advisable to use simple and everyday language while informing patients of ethnic minorities. Undoubtedly, determining the reason behind patients’ hesitation is important to guide them to quickly make decisions.

In terms of medical insurance, patients who had URBMI more frequently had delayed informed consent. Patients with URBMI are urban residents, indicating that their socioeconomic level and understanding of disease might be better than that of patients with NRCMS, who are more rural. As a result, patients with URBMI may have a strong sense of self-determination and prefer to spend time thinking independently. In addition, medical paternalism, in which priority is given to the physician’s decision, is more prevalent among rural populations. Informed consent procedures involve patients and medical personnel, and shared decision-making is ideal for both parties; emergency care patients are in even greater need of physicians’ expertise. The present findings implicate that physicians should respect patients’ autonomy and join in decision-making rather than be a complete bystander or dominator.

Moreover, our results suggested that intermittent chest pain and chest pain relief were positively associated with the informed consent delay time. Because these are atypical symptoms of STEMI that do not seem to critically endanger patients, they might be considered less risky. In fact, patients with life-threatening manifestations require immediate emergency treatment, and preparation for such treatment could be started early before the informed consent signature is obtained. Thus, patients and their relatives tend to spend more time evaluating risks and giving consent. This finding is of practical significance in the sense that regardless of the severity of the disease, early reperfusion increases treatment effectiveness; it also provides suggestive evidence that physicians should help patients to recognize the diversity of STEMI manifestations and the benefits of timely reperfusion while providing medical information to the patients or their relatives.

Compared with walk-in, other transport modes significantly lengthened the informed consent delay time. A possible explanation is that patients who arrive at the hospital by EMS may have a relatively serious condition, resulting in a longer discussion to assess the risk of PCI therapy. Patients who are transferred to hospitals have already undergone the process of discharge and readmission. Such patients have already experienced a long period of early delay and a high cost of time and making critical decisions,^[26,27]^ which may lead to hesitations in signing informed consent. Additionally, because these patients’ symptoms are more critical and acute, the overall delay time is much longer once a delay in obtaining informed consent has occurred given that patients and their relatives need more time to evaluate the risks and give consent. The situations of patients with in-hospital onset are more complex because they might have comorbidities and complications. This requires them to sign multiple informed consent forms, prolonging doctor–patient communication and slowing obtainment of the signature.

Another principal finding is that informed consent delay was significantly associated with prolongation of the door-to-balloon time, which potentially led to poor clinical outcomes.^[5,8,28,29]^ This finding is compatible with other studies.^[11,25,30]^ Shavadia *et al*.^[28]^ reported that every 10-minute delay in initiating catheterization was correlated with an increase in the door-to-device time, which lengthened the door-to-balloon time far beyond the recommended time of 90 min. This would inevitably give rise to a longer delay of PCI because with the postponement of consent, patients’ condition might worsen, resulting in a longer time required to complete PCI. This highlights the importance of the association between the informed consent time and preactivation of the catheterization laboratory. Nonetheless, follow-up prospective randomized studies are warranted to confirm the net effect of informed consent delay on clinical outcomes.

In clinical practice, Chinese doctors are merely responsible for the provision of medical information, whereas patients are left alone to make decisions. However, because of patients’ poor understanding of medical information and fragile trust in clinicians or medical institutions, patients commonly hesitate to make decisions.^[17,31]^ Thus, medical workers should pay attention to their communication skills and avoid medical terminology in the transfer of knowledge. Physicians’ expertise, empathy, and respect for patients may help to build trust between them.^[17]^ Additionally, clinicians should join patients in decision-making, help patients quickly understand the emergency and its risks, and induce patients to make an optimal choice within a limited time. To improve the informed consent procedure, it is advisable to give more weight to humanistic training in medical education, such as communication skills and professionalism.^[14]^

Several limitations of this study should be acknowledged. First, because of limited access to patients’ information, we did not analyze other sociodemographic factors such as economic status and educational attainment. However, previous qualitative studies have investigated the contribution of these elements to informed consent delay. Additionally, we believe that the data available in the Database were maximally utilized to predict the factors related to informed consent delay. Second, although underage patients (< 18 years of age) constitute a certain proportion and may exert an influence on the results, they were excluded from the study. The informed consent procedure of underage patients is rather complex because their relatives can be surrogate signers. Their condition requires a separate discussion. Finally, regional differences in factors associated with a delayed informed consent time were not evaluated within our analysis. Nevertheless, our study employed generalized linear mixed models with a random-effect term for hospitals across geographic regions to account for clustering of hospitals, which may have minimized the bias resulting from disparities in regions and thus ensuring the reliability of our findings.

## Conclusion

Informed consent delay is significantly associated with the door-to-balloon time which plays a crucial role in achieving better outcomes for patients with STEMI. It is essential to shorten the delay time caused by an extended informed consent procedure. This can probably be achieved by identifying and addressing modifiable factors that are associated with shortening the informed consent procedure in China and other countries, in which performing a complete informed consent procedure is necessary for any emergency.
